# Molecular subtypes identified by pyroptosis-related genes are associated with tumor microenvironment cell infiltration in colon cancer

**DOI:** 10.18632/aging.204379

**Published:** 2022-11-16

**Authors:** Yiting Ling, Yinda Wang, Chenxi Cao, Lianzhong Feng, Binzhong Zhang, Senjuan Li

**Affiliations:** 1 Department of Anorectal Surgery, Jiaxing Second Hospital, Jiaxing 314000, Zhejiang, P.R. China; 2 Department of Gastrointestinal Surgery, Jiaxing Second Hospital, Jiaxing 314000, Zhejiang, P.R. China

**Keywords:** pyroptosis, prognosis, immune response, tumor microenvironment, immunotherapy

## Abstract

The important role of pyroptosis in tumor progression has been well characterized in recent years. However, little is known about the impact of tumor pyroptosis characteristics on patient prognosis and tumor microenvironment (TME) as well as efficacy of immunotherapy. In this study, we successfully classified colon cancer samples into three pyroptosis characterizations with different prognosis and TME cell infiltration patterns based on the expression of pyroptosis-related genes. Cluster 2, with the characterizations of immunosuppression, was classified as immune-desert cell infiltration patterns. Cluster 3, with the patterns of immune-inflamed cell infiltration, had the feature of an activated innate and adaptive immunity and significant prolonged survival. The activation of stromal pathways including EMT, angiogenesis and TGF-β in cluster 1 may mediate the impaired immune penetration of this cluster, which was classified as immune-excluded cell infiltration patterns. Our results demonstrated the PyroSig signature was a robust and independent biomarker for predicting patient prognosis. Patients with low PyroSig signature was confirmed to be correlated with treatment advantages and significant prolonged survival in two anti-checkpoint immunotherapy cohorts. This study identified three pyroptosis-related subtypes with distinct molecular features, clinical and microenvironment cell infiltration patterns in colon cancer, which could promote individualized immunotherapy for colon cancer.

## INTRODUCTION

As one of the most common tumor types in digestive system, the incidence rate and mortality of colon cancer are increasing year by year [1–3]. Although in recent years, the comprehensive treatment mainly including surgical resection, chemotherapy, and radiotherapy has enriched the treatment methods of colon cancer, the prognosis of patients has not been significantly improved, and the 5-year survival rate is 40% - 60% [4, 5]. The amazing benefits of immunotherapy represented by PD-1/PD-L1 immune checkpoint inhibitors on a variety of solid tumors have provided the new direction and strategy for the treatment of colon cancer [6, 7]. Unfortunately, anti-PD-1/PD-L1 therapy did not show the desired therapeutic effect in patients with colon cancer. Only 10% - 20% of patients could benefit from the anti-PD-1/PD-L1 therapy, which was far from meeting the clinical needs [8, 9]. Significant individual heterogeneity especially the existed intrinsic and adaptive immune resistance in tumor microenvironment (TME) was significantly associated with treatment failure. Therefore, it is urgent to develop new biomarkers to predict the prognosis of patients and determine the treatment regimens. Increasing evidence indicated that the pyroptosis played a crucial role in the initiation and progression of colon cancer via multiple biological pathways. Pyroptosis, a distinct form of programmed cell death distinguished from apoptosis, is characterized by cell swelling and rupture, releasing inflammatory cellular contents, which triggers a robust inflammatory response [10, 11]. A variety of cytokines and stress-related signaling molecules are activated concomitantly with the process of pyroptosis, which promotes immune cell infiltration as well as inflammatory responses. During pyroptosis, activated caspase-1 promotes the production of proinflammatory cytokines such as IL-18 an IL-1β7 to regulate the tumor immune microenvironment. Furthermore, since pyroptosis is an innate immune mechanism, it could likewise suppress tumor progression [12–15]. However, the TME cell infiltrating patterns mediated by distinct pyroptosis characteristics in colon cancer remains unknown. Exploring the impact of distinct pyroptosis characteristics on immune cells of the TME will help to increase the understanding of the complexity and heterogeneity of the TME and provide novel directions and strategies for personalized immunotherapy for colon cancer patients. In the present study, we performed genomic analyses to comprehensively assess the pyroptosis characterizations in colon cancer based on the expression of pyroptosis-related genes, and correlated these characterizations with TME immune cell infiltration patterns. Three pyroptosis characterizations with distinct prognostic features and TME cell infiltration patterns were successfully identified in colon cancer based on more than 1000 cases. The constructed pyroptosis-related score signature (PyroSig) could serve as an independent biomarker to predict patient prognosis and efficacy of anti-PD-1/PD-L1 immunotherapy.

## RESULTS

### Genomic variation of pyroptosis-related genes in colon cancer

**Figure 1:**
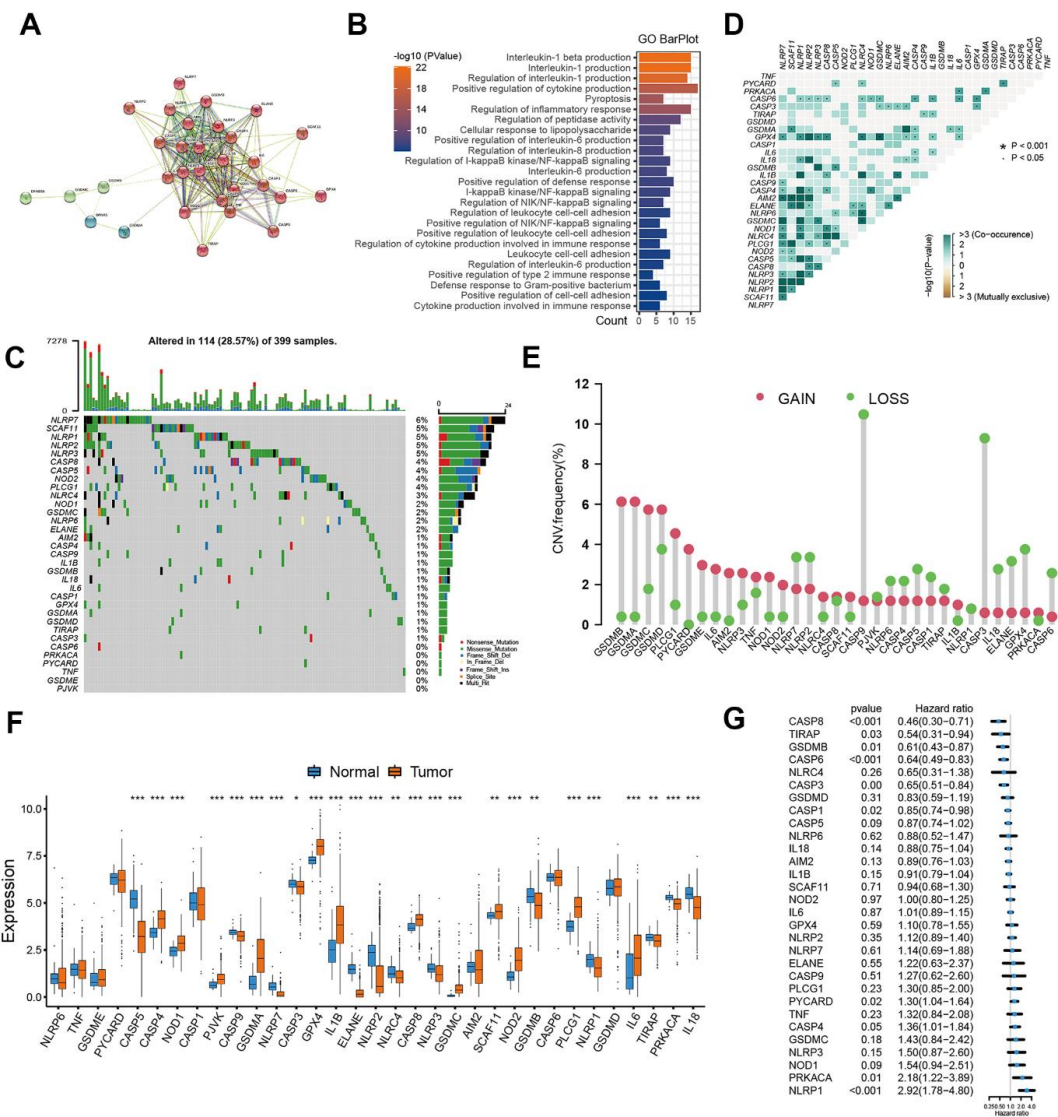
**Landscape of pyroptosis related genes in colon cancer.** (**A**) The protein-protein interactions (PPI) network between pyroptosis genes using STRING database. (**B**) GO functional enrichment analyses for pyroptosis-related genes. (**C**) The mutation landscape of pyroptosis genes in TCGA-COAD cohort. (**D**) The mutation co-occurrence and exclusion analyses for these genes. (**E**) The copy number variation frequency of pyroptosis genes. (**F**) Expression of pyroptosis genes in tumor and normal samples based on TCGA-COAD cohort. (**G**) Survival analyses for the pyroptosis genes using univariate Cox regression model.

Based on the published studies, we in total extracted 31 pyroptosis-related genes from the GEO datasets. We used the STRING to establish the protein-protein interaction network (PPI), and reveal widespread protein interactions among these genes (Figure 1A). The GO enrichment analysis was used to reveal the biological functions of these genes, and we found these genes were remarkably related to pyroptosis and immune regulation pathways including pyroptosis, nterleukin-1 production and cytokine production involved in immune response (Figure 1B). only 114 of 399 samples, accounting for 28.57%, exhibited at least one mutation type among these genes (Figure 1C). We also observed an obvious mutation co-occurring relationship existed among these genes (Figure 1D). In addition, the CNV alteration analyses showed that these pyroptosis-related genes had a relatively low CNV alteration frequency in colon cancer. GSDMB, GSDMA, GSDMC, PLCG1, PYCARD, IL6, AIM2 and GSDME primarily focused on copy number amplification frequencies, whereas CASP9, CASP3, IL18, ELANE, GPX4 and CASP6 had copy number deletions (Figure 1E). For the mRNA expression, a significant difference in expression was observed between tumor and normal tissues (Figure 1F). Considering the difference in expression, we performed survival analyses for these pyroptosis-related genes, and the univariate Cox regression model indicated these genes were significantly associated with prognosis of patients, of these, NOD1, PRKACA and NLRP1 were the protective factors for colon cancer (Figure 1G).

### Identification of distinct pyroptosis characterization clusters

**Figure 2:**
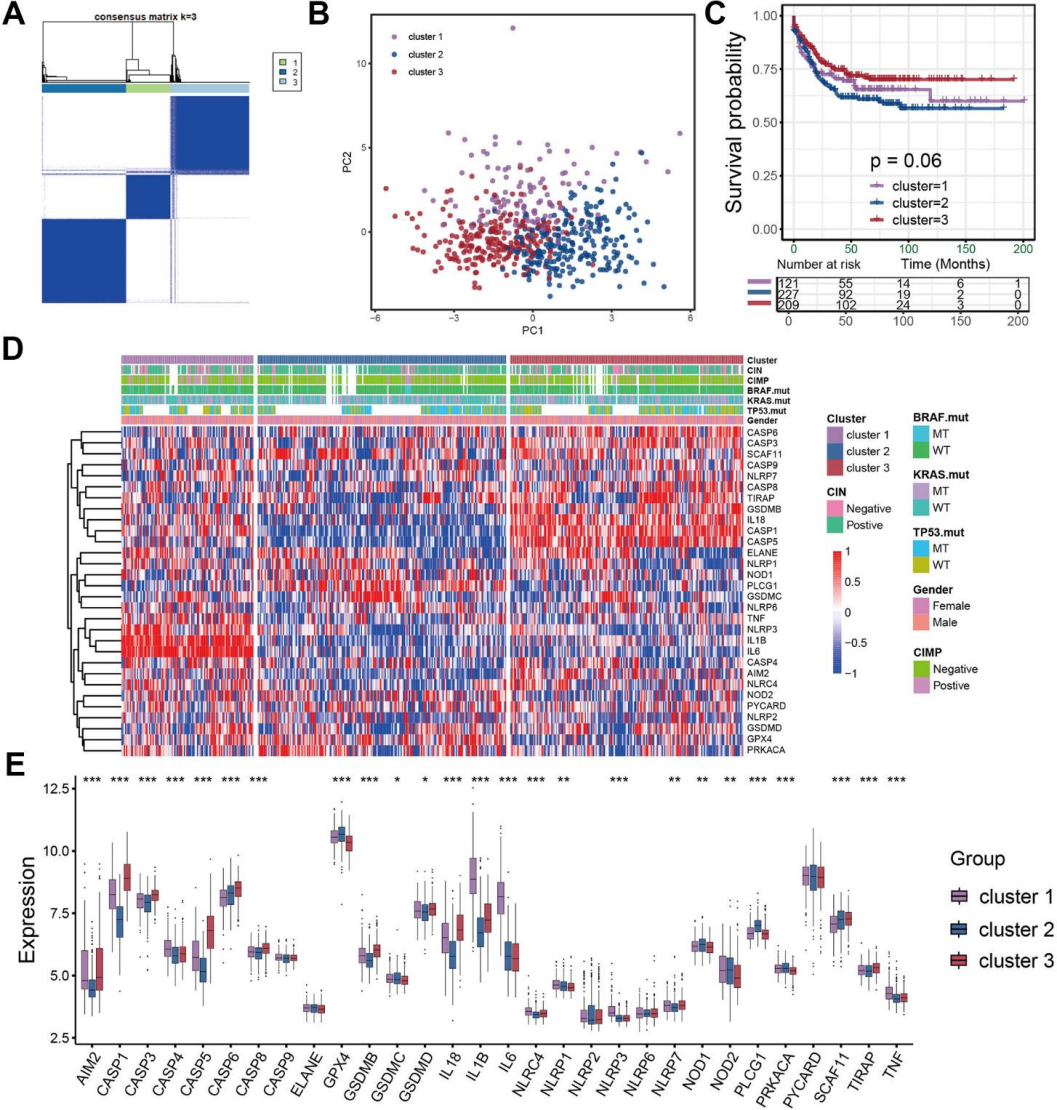
**Identification of distinct pyroptosis characterization clusters in colon cancer.** (**A**) Consensus matrices of pyroptosis related genes, for k=3. (**B**) Principal component analysis for the three clusters based on the pyroptosis gene expression and revealed three entirely disjoint populations in the meta cohort. (**C**) Survival analyses for the three pyroptosis characterizations including cluster 1, cluster 2 and cluster 3 in the meta cohort. (**D**) The hierarchical clustering of pyroptosis genes among three pyroptosis characterization clusters. (**E**) Difference in pyroptosis gene expression between the three clusters.

Considering the detailed clinical annotations in GSE39582 cohorts, we used this cohort for the subsequent analyses. The consensus clustering was utilized to reveal the pyroptosis characterization in colon cancer and classify patients into distinct molecular subtypes. Based on the expression of entire pyroptosis-related genes, we successfully classified all samples into three different pyroptosis characterizations, which was referred to as cluster 1 subtype, cluster 2 subtype and cluster 3 subtype, respectively (Figure 2A). PCA methods presented three completely disjoint populations among three clusters (Figure 2B). Among three subtypes, patients classified as cluster 2 subtype experienced a poor survival, while patients with cluster 3 subtype showed a particularly prominent prolonged survival (Figure 2C). The significant distinction in all pyroptosis-related gene expression was observed among the three clusters (Figure 2D, 2E). In these three pyroptosis characterizations of colon cancer, we found CASP4, GSDMC, IL1B, IL6, NLRC4, NLRP1, NLRP3 and TNF was significantly up-regulated in cluster 1 subtype compared to other two subtypes. This implied that pyroptosis in this cluster was mainly regulated by CASP4, IL1B, IL6, NLRC4, NLRP1, NLRP3 and TNF. The pyroptosis characterization characterized by high expression of this set of genes mediated the immune-excluded phenotype of tumor microenvironment, which led to a relatively poor prognosis. Similarly, cluster 2 subtype presented an upregulation expression of GPX4, GSDMC, NOD1 and PLCG1, whose pyroptosis characterization, regulated by this gene set, led to an immune-desert microenvironment. And cluster 3 subtype showed a high expression of AIM2, CASP1, CASP3, CASP5, CASP6, CASP8, GSDMB, IL18 and TIRAP, whose pyroptosis characterization, regulated by this gene set, resulted in an improved prognosis and immune-inflamed tumor microenvironment (Figure 2D, 2E).

### TME cell infiltration patterns under three pyroptosis characterizations

**Figure 3:**
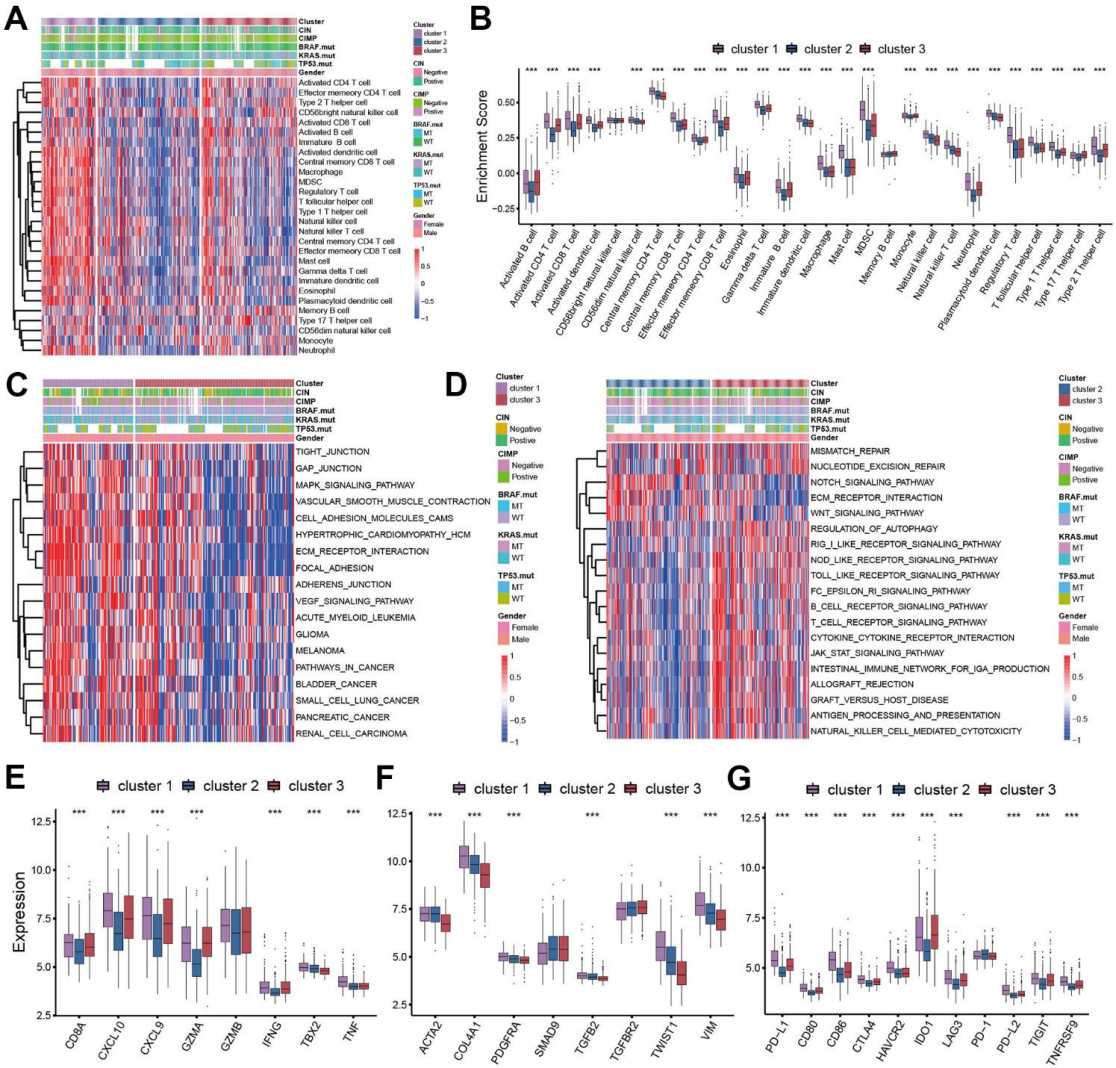
**TME cell infiltration patterns in distinct pyroptosis characterizations.** (**A**) The abundance of 28 TME cell infiltration among three pyroptosis characterization clusters visualized by heatmap. (**B**) Differences of 28 TME cell infiltration abundance between three pyroptosis characterization clusters. (**C**, **D**) GSVA enrichment showing the activation states of biological pathways in distinct clusters. (**C**) cluster 1 vs cluster 3; (**D**) cluster 2 vs cluster 3. (**E**) Difference in the immune-activation related gene expression among three clusters. (**F**) Difference in the TGFβ-EMT pathway-related gene expression among three pyroptosis characterization clusters. (**G**) Difference in the immune-checkpoint related gene expression among three clusters.

In order to evaluate the correlation between TME cell infiltration patterns and pyroptosis characterizations, we calculated the infiltration abundance of immune cells in each cluster. We found a high level of immune cell infiltration in the tumor microenvironment of cluster 1, sharing the similar immune cell infiltration abundance with the cluster 3 (Figure 3A, 3B). However, despite the high immune level of cluster 1, we did not observe that patients with cluster 1 showed significant survival advantages compared with the other two subtypes (Figure 2C). We then applied the GSVA enrichment analysis to explore this phenomenon by revealing the biological behaviors under these distinct three clusters. As shown in Figure 3C, 3D, we noticed that the immune related signaling pathway was significantly activated in cluster 3 subtype such as cytokine-cytokine receptor interaction, Toll like receptor signaling pathway, T cell receptor signaling pathway, and Nod like receptor signaling pathway (Figure 3D). The activity of stromal-related and carcinogenic pathways was remarkably up-regulated in the cluster 1 subtype including ECM receptor interaction and focal adhesion (Figure 3C). While in cluster 2 subtype, we found the immunosuppressive pathways were significantly activated, which was consistent with the results of immune cell infiltration.

**Figure 4:**
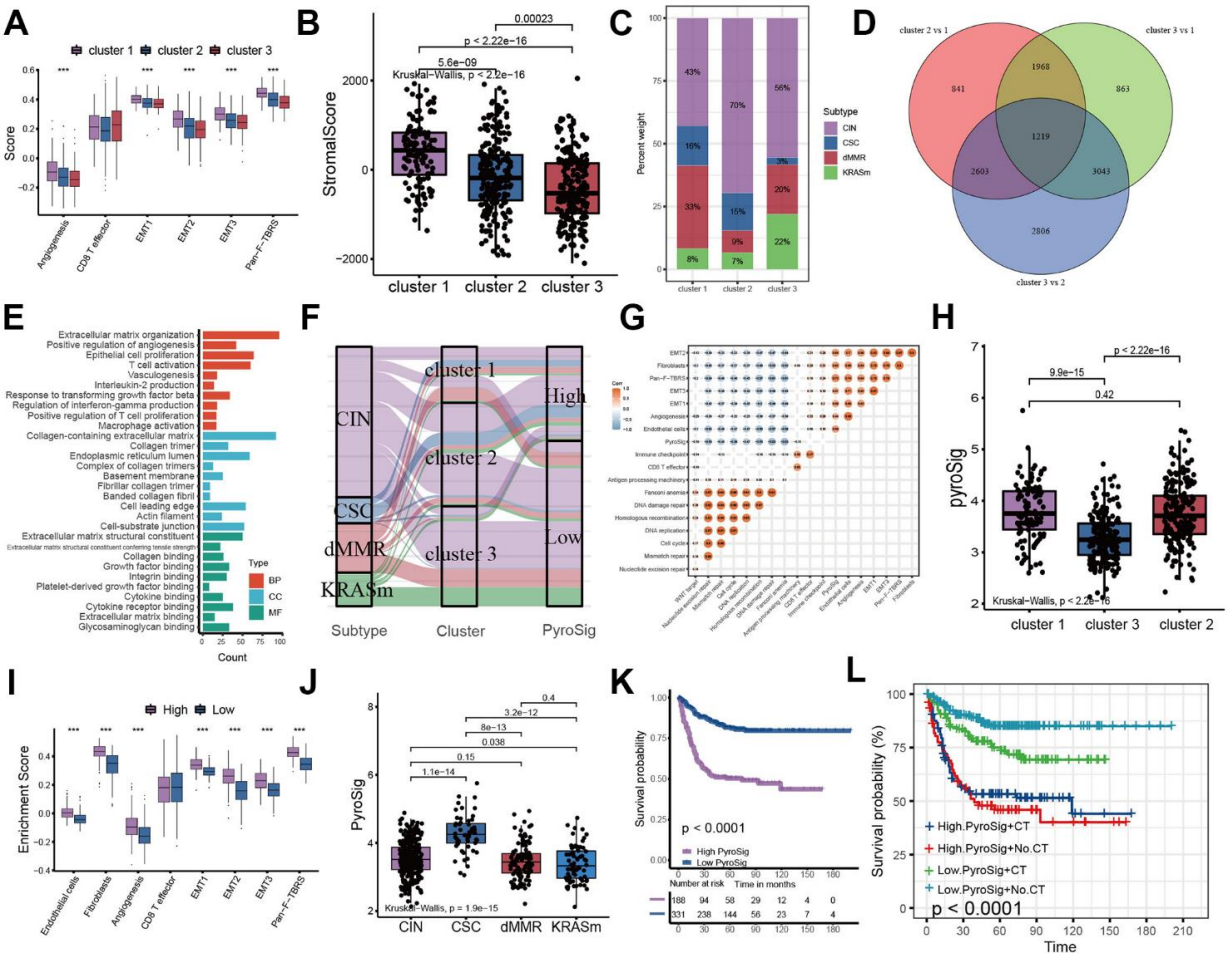
**Construction of pyroptosis related score signature (PyroSig).** (**A**) Variations between three distinct pyroptosis characterization clusters in pathways with stroma activation. (**B**) ESTIMATE algorithm analyses revealing the overall TME stromal score among three clusters. (**C**) The proportion of molecular subtypes in the three clusters. (**D**) The venn diagram showing 1219 overlap DEGs between three clusters. (**E**) GO functional enrichment analyses for overlap DEGs. (**F**) The changes of molecular subtypes, pyroptosis characterization clusters and PyroSig, visualized by alluvial diagram. (**G**) Spearman correlation between the known signatures and PyroSig values. (**H**) Differences in PyroSig signature across three distinct pyroptosis characterization clusters. (**I**) Difference of stromal signature between low and high PyroSig groups. (**J**) Differences in PyroSig across distinct molecular subtypes. (**K**) Kaplan-Meier curves showing the survival difference between the low and high PyroSig groups. (**L**) Survival analyses of four subgroups, where patients were stratified according to adjuvant chemotherapy.

We analyzed the expression of chemokines and cytokines that characterize these three pyroptosis characterization clusters to further explore their characteristics of TME immunoregulation (Figure 3E–3G) [16]. The results indicated that the mRNA expression of TGFb/EMT pathway was significantly upregulated in cluster 1 subtypes (Figure 3F), and the cluster 1 and cluster 3 subtypes demonstrated increased expression of mRNAs associated with immune-activation transcripts (Figure 3E). In addition, the cluster 3 subtypes showed an upregulated expression of immune checkpoint molecules, which may mediate the immune escape of this subtype (Figure 3G). Based on the above results, we were surprised to find that the three pyroptosis characterizations of colon cancer were consistent with the three immunophenotypes of the tumor microenvironment, which all shared similar immune cell infiltration patterns. The immune cell infiltrate characteristic of cluster 1 subtype was consistent with the immune-excluded phenotype, which was characterized by marked stromal activation of the tumor microenvironment. The cluster 2 subtype was consistent with an immune-desert phenotype, with a microenvironment with little immune cell infiltration. Whereas the tumor microenvironment of cluster 3 subtype phenocopies a relatively high level of infiltration of innate and adaptive immune cells, which was consistent with an immune-inflamed phenotype. We then used the gene signature to further evaluate the classification accuracy. In order to further validate the stability of these findings, we used the ssGSEA to estimate the activity of stromal pathways through stromal signature, and found the activation of angiogenesis pathways, pan-fibroblast transforming growth factor beta response (Pan-F-TBRS), and epithelial-mesenchymal transition (EMT) (Figure 4A). The ESTIMATE algorithm showed the stromal score was significantly higher in cluster 1 subtype compared with other two subtypes (Figure 4B). Subsequent analysis revealed that the majority of patients with a molecular phenotype of CIN presented with the pyroptosis characterizations of cluster 2, whereas the majority of patients with a dMMR molecular subtype presented with the pyroptosis characterizations of cluster 1 (Figure 4C, 4F). The above analyses also demonstrated that TME cell infiltration patterns under the three pyroptosis characterizations were in accordance with three immunophenotypes of tumor including immune-inflamed, immune-excluded and immune-desert phenotypes, indicating the accuracy of our classification.

### Construction of pyroptosis related signature (PyroSig)

To further explore the potential biological characteristics of the three pyroptosis characterizations, we investigated the transcriptome differences between the three clusters and a total of 1219 DEGs were determined (Figure 4D). We analyzed differentially expressed genes (DEGs) common to the three subtypes to uncover biological pathways that differ among subtypes. A total of 1219 DEGs were identified, and we found that these DEGs were similarly enriched for immune-related signaling pathways, confirming again that pyroptosis characterizations in colon cancer were significantly correlated with TME anti-tumor immune response (Figure 4E). We established the PyroSig signature by the LASSO analysis to further evaluate the role of pyroptosis characterizations in patient survival and TME cell-infiltrating patterns. The coefficient of each signature gene was summarized in Supplementary Table 2 and Supplementary Figure 1. We used the MaxStat algorithm to classify patients into low and high PyroSig groups. The flow of samples including molecular subtypes, pyroptosis clusters and PyroSig was presented by the alluvial diagram (Figure 4F). The PyroSig signature was negatively correlated with DNA damage repair and was positively correlated with stromal activity (Figure 4G). The cluster 1 and 2 pyroptosis characterization displayed the highest median PyroSig, while the cluster 2 showed the lowest median PyroSig (Figure 4H). The stroma in patients with high PyroSig was remarkably activated compared with that in patients with low PyroSig (Figure 4I). The CSC molecular subtype exhibited a highest PyroSig compared to the other three molecular subtypes (Figure 4J). Patients with low Pyrosig scores were associated with significantly prolonged survival (Figure 4K). Furthermore, we found that the Pyrosig signature could similarly be used to predict survival in patients who received adjuvant chemotherapy. Patients with lower Pyrosig were consistently associated with improved survival regardless of whether or not patients received adjuvant chemotherapy. (Figure 4L).

### Characteristics of PyroSig in tumor somatic mutation

**Figure 5:**
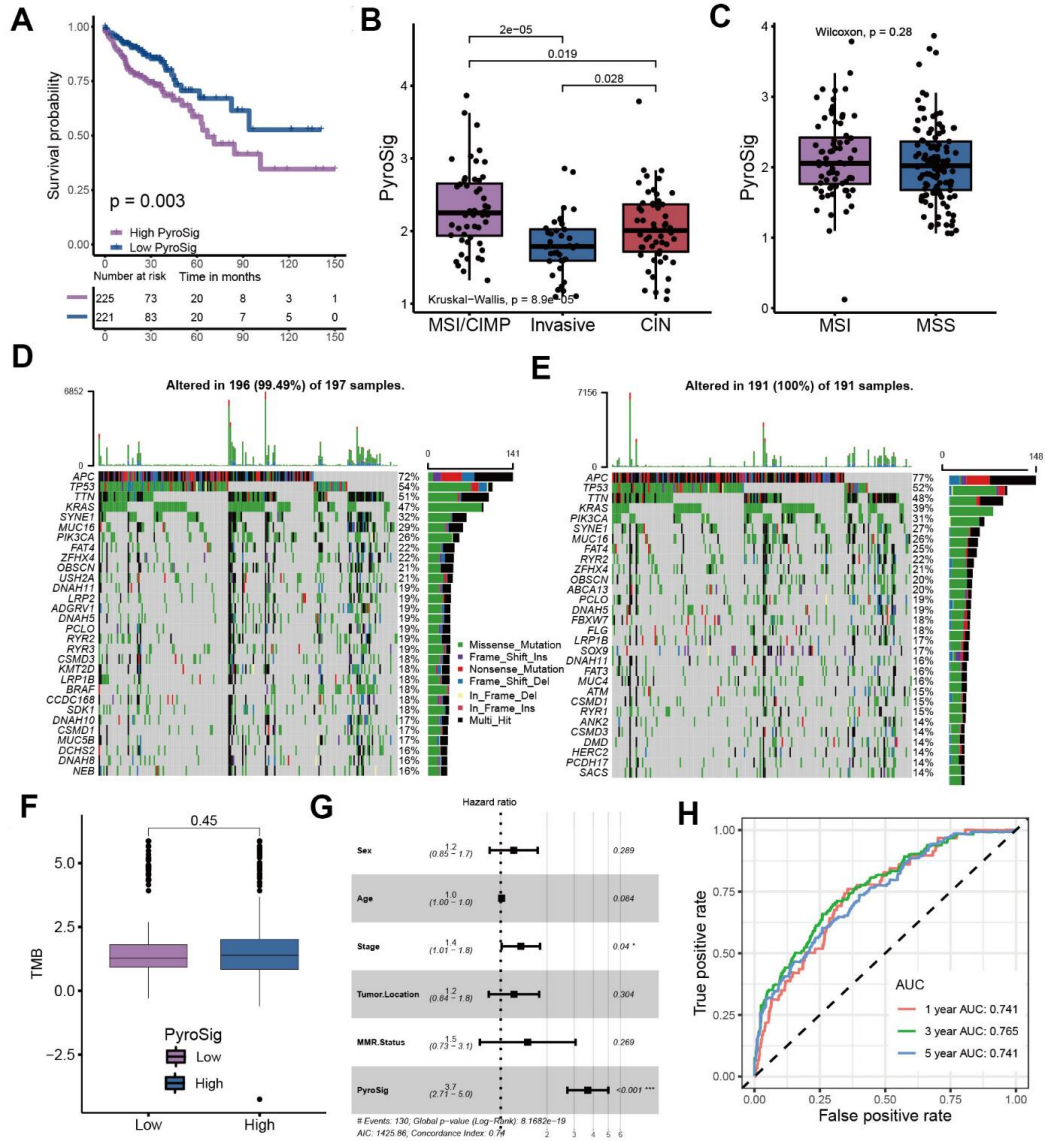
**Characteristics of PyroSig in tumor somatic mutation.** (**A**) Kaplan-Meier curves showing the survival analyses of high and low PyroSig groups in TCGA-COAD cohort. (**B**) Differences in PyroSig between distinct TCGA colon cancer molecular subtypes. (**C**) Differences in PyroSig between different microsatellite status. (**D**, **E**) The waterfall plot showing the differences of TMB landscape between low and high PyroSig groups. (**D**) High PyroSig group. (**E**) Low PyroSig group. (**F**) Differences in tumor burden mutation between low and high PyroSig groups. (**G**) Multivariate cox regression analysis for PyroSig in predicting patient’s survival. (**H**) ROC curves showing the predictive values of PyroSig in prognosis.

We used TCGA-COAD cohort as the validation set. Consistent with the GEO cohort, patients with low PyroSig also experienced an improved overall survival compared to those with high PyroSig (Figure 5A). Compared with CIN and invasive molecular subtype, patients with MSI/CIMP exhibited a relatively higher PyroSig (Figure 5B). However, we found MSI and MSS phenotype did not show an obvious difference (Figure 5C). We then explore the somatic mutation landscape of the high and low PyroSig group to reveal the correlation between tumor mutation burden and PyroSig (Figure 5D–5F). The tumor mutation burden (TMB) in tumors with low PyroSig were similar with those with high PyroSig (Figure 5F). We plotted the frequency of mutations as well as the types of mutations in the top 30 mutated genes between high and low PyroSig group using waterfall plots (Figure 5D, 5E). Based on the multivariate Cox regression model consisting of sex, age, tumor location and MMR status, we confirmed that the PyroSig was an independent and robust prognostic biomarker to predict the outcomes of patients with colon cancer (Figure 5G). Additionally, the predictive performance of PyroSig signature to evaluate the one, three and five year survival rates, confirmed by the ROC curves, reached 0.741, 0.765 and 0.741, whose reliability was far superior to the traditional pathological evaluation (Figure 5H).

### Predictive performance of PyroSig in anti-PD-1/PD-L1 immunotherapy

**Figure 6:**
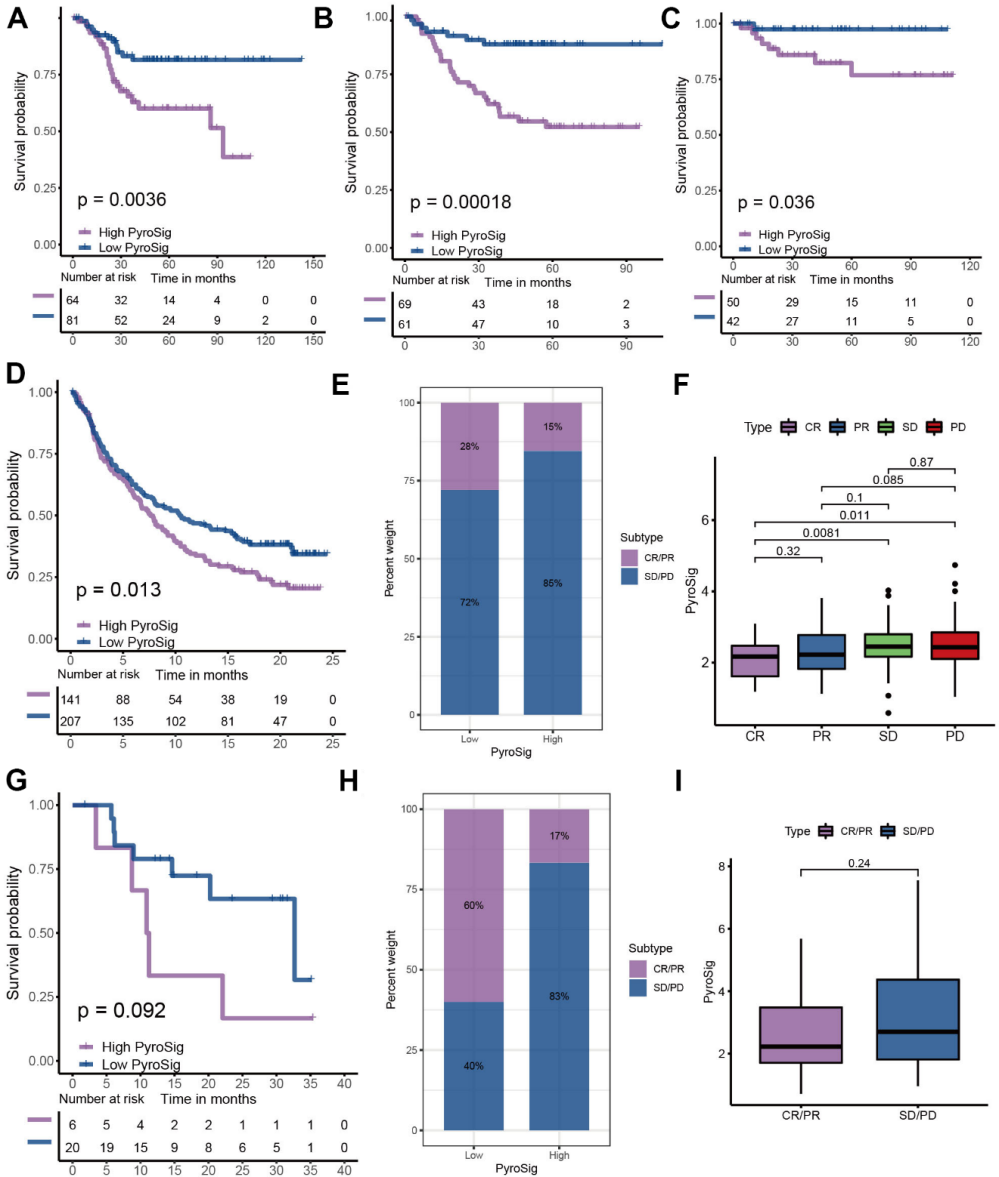
**Role of PyroSig in predicting efficacy of immunotherapy.** (**A**) Kaplan-Meier curves showing the survival analyses of high and low PyroSig groups in GSE17536 cohort. (**B**) Kaplan-Meier curves showing the survival analyses of high and low PyroSig groups in GSE37892 cohort. (**C**) Kaplan-Meier curves showing the survival analyses of high and low PyroSig groups in GSE38832 cohort. (**D**) Kaplan-Meier curves displaying the survival difference of high and low PyroSig groups in IMvigor210 cohort. (**E**) The ratio of clinical response types in high PyroSig and low PyroSig groups in the IMvigor210 cohort when treated with anti-PD-1 immunotherapy. (**F**) Differences in PyroSig between different clinical response types in the IMvigor210 cohort. (**G**) Survival analyses for PyroSig in GSE78220 anti-PD-1 immunotherapy cohort. (**H**) The ratio of clinical response types in high PyroSig and low PyroSig groups in the GSE78220 cohort when treated with anti-PD-1 immunotherapy. (**I**) Differences in PyroSig between different clinical response types in the GSE78220 cohort.

To further determine the prognostic predictive value of the PyroSig signature, we generalized it to other colon cancer cohorts. Based on the GSE17536, GSE37892 and GSE38832 collected from GEO database, we found that patients with lower PyroSig were consistently associated with improved survival compared with those with higher PyroSig, which further confirmed the potential of PyroSig signature as an independent prognostic biomarker in colon cancer (Figure 6A–6C). The successful application of immunotherapy on multiple solid tumors, represented by immune checkpoint inhibitors, has provided a new strategy for the comprehensive treatment of colon cancer. In this study, we collected two immunotherapy cohorts to evaluate the role of PyroSig signature in predicting the efficacy of anti-PD-1/PD-L1 immunotherapy. In the IMvigor210 cohort, which investigated the efficacy of anti-PD-L1 regimens, we found the patients with low PyroSig presented a prominent improved clinical response and survival than patients with high PyroSig (Figure 6D–6F). Consistent with anti-PD-L1 regimens, PyroSig signature could be also used to predict the outcomes of patients treated with anti-PD-1 regimens (Figure 6G, 6H). The above results showed a potential predictive value of PyroSig in anti-PD-1/PD-L1 immunotherapy.

## DISCUSSION

Although the diagnosis and treatment of colon cancer have made some progress in recent years with the development of technology, it has not significantly reduced the incidence rate and mortality of colon cancer. In addition, for advanced colon cancer, existing treatment methods are still limited. Therefore, the early diagnosis and treatment as well as the mechanism of recurrence monitoring are particularly important in colon cancer [17]. Due to the individual heterogeneity of colon cancer, some clinical indicators, such as age, stage, image characteristics and blood biomarkers, have limited effect in predicting prognosis and evaluating treatment plans. In recent years, with the wide application of large-scale sequencing technology, evaluating the gene expression level and mutation characteristics has become one of the important means for prognosis monitoring of many solid tumors. However, a single gene expression level is easily affected by various factors *in vivo*, and its prediction accuracy is often poor. It has become an important means to improve the prediction efficiency to identify novel molecular subtypes and build a multi gene model by combining a group of gene signature with machine learning [18]. Pyroptosis plays a dual role of promoting or anti-tumor in mediating inflammation and tumor progression [19–21]. On the one hand, inflammasomes have the function of eliminating microorganisms and maintaining the integrity of intestinal epithelium, which can prevent tumor attraction. In contrast, inflammasomes can also stimulate the production of protective factors of cnacer cells to help cancer cells escape immune killing, in which, immunosuppressive factors such as Il-18 and il-1 β accumulate in the tumor microenvironment, impairing the function of natural killer immune cells and mediating the immunosuppressive microenvironment [22]. Yang et al. reported that cisplatin exerted antitumor roles in triple-Negative Breast Cancer through promoting MEG3/NLRP3/caspase-1/GSDMD pathway to mediate pyroptosis [23]. Zhang et al. revealed that GSDME silencing remarkably suppressed cisplatin induced secondary necrosis/pyroptosis, but could not inhibit paclitaxel induced secondary necrosis/pyroptosis [24]. At the same time, the antineoplastic features of pyroptosis have been widely demonstrated, possibly due to the protective effect of inflammasomes on the gastrointestinal epithelium. However, the TME cell-infiltrating patterns mediated by different pyroptosis characteristics in colon cancer remains unknown. In this study, we comprehensively evaluated the molecular characteristics of genes related to pyroptosis to construct new classifiers and prognostic signature, and associated them with immune cell infiltration in the tumor microenvironment of colon cancer, so as to guide more appropriate treatment strategies.

Here, we integrated multi-omics data on colon cancer from the TCGA database to further explore the genomic signatures of cell-coke-death-related genes in colon cancer tissues. Although the copy number variation and mutation frequency of these genes are relatively low, the expression levels of these genes are significantly different between colon cancer tissues and normal tissues. Through consensus clustering of these genes, we revealed three distinct cellular pyroptosis characterizations, implying that colon cancer patients could be divided into three distinct molecular subtypes based on pyroptosis-related genes. GSVA and GO enrichment analysis showed that these three subtypes were highly correlated with immune related signaling pathways. The evaluation of TME immune cell infiltration confirmed that these three pyroptosis related molecular subtypes had significantly different TME cell infiltration patterns, and were consistent with the three immune phenotypes of tumors. In general, innate immune cells and stroma were significantly activated in cluster 1 subtypes, which was consistent with the immune-excluded phenotype. Cluster 2 subtype presented an inhibitory microenvironment with relatively few innate and adaptive immune cells, so it was classified as immune-desert phenotype. Contrary to the former two, the microenvironment in cluster 3 subtype had a large number of immune cells infiltrating, and the immune related signal pathway also showed an activated phenotype, which was consistent with the immune-inflamed phenotype. Although both cluster 1 and cluster 3 subtypes belong to “cold tumors”, their mechanisms were not identical. The stroma in the TME was significantly activated in cluster 1 subtype, mediating tumor immune escape. There were abundant immune cells in both immune-excluded and immune-inflamed tumors. However, unlike the immune-inflamed tumors, the activated stroma retained immune cells around the nests of tumor cells, limiting immune cell infiltration into the parenchyma of the tumor. The interaction between the stroma and the immune cells made the immune cells appear to be present inside the tumor. Although PD-1/PD-L1 inhibitors could stimulate T cell activation and proliferation around the stroma, they could not stimulate infiltration, thus limiting the clinical response rate of this subtype to immunotherapy [25–29]. In addition, activation of the classical oncogenic pathway in cluster 1 subtype further reduced the level of immune cell infiltration [30]. Our results suggested that impaired immune permeability of the cluster 1 subtype may be associated with activation of EMT, TGF-β, and angiogenic pathways.

At present, it has become a hot topic to transform “cold tumor” into “hot tumor” to increase immune infiltration of microenvironment [31–33]. In this study, we identified three molecular subtypes closely related to the immune phenotype through pyroptosis. This suggested that pyroptosis may be a factor that could not be ignored in mediating the complexity of immune infiltration in microenvironment. Regulating pyroptosis-related genes to change the pyroptosis characteristics may be a potential strategy to reshape the microenvironment. We used LASSO algorithm to construct PyroSig signature to further characterize the role of pyroptosis in prognosis and TME cell infiltration patterns. Cluster 3 subtypes were characterized by low PyroSig signature, while cluster 1 and cluster 2 subtypes were characterized by high PyroSig signature. Subsequent analysis revealed that PyroSig signature could be used as an independent biomarker to predict the prognosis of colon cancer patients. We also found that pyroptosis could be involved in the patient's resistance to immune checkpoint immunotherapy. In patients receiving anti-PD-1 and anti-PD-L1 immunotherapy, low PyroSig was closely associated with enhanced clinical response and significantly prolonged survival. Although our results indicated the pyroptosis-related signature could be associated with efficiency of immunotherapy, due to the lack of single-cell sequencing data related to pyroptosis and immunotherapy, we were unable to further determine which cell subset of pyroptosis mediated the efficacy of immunotherapy. Determining which cell subset of pyroptosis played a crucial role in immunotherapy could help to further guide more effective immunotherapeutic strategies. This was a potential limitation of our study.

## CONCLUSIONS

This study identified three pyroptosis characterizations with distinct clinical, molecular characteristics and TME cell infiltration patterns, and constructed PyroSig signature based on these pyroptosis characterizations, which could be served as a robust and independent biomarker for predicting patient outcomes and efficacy of immunotherapy. It might help to promote individualized immunotherapy for colon cancer from the perspective of pyroptosis characterizations.

## MATERIALS AND METHODS

### Sample datasets collection and processing

After systematic search of GEO and TCGA database, we collected a total of 5 colon cancer cohorts including the TCGA-COAD, GSE39582, GSE17536, GSE37892, GSE38832, which contain detailed clinical information [34–37]. All cohorts from the GEO database were based on the Affymetrix platforms, so we used the affy R package for background correction and normalization [38]. For TCGA-COAD cohort, we downloaded the FPKM value of the original gene expression and converted FPKM into transcripts per kilobase million (TPM) values. We merged the GEO cohorts and used the sva R package to perform batch correction. The detailed clinical information of all the included cohorts was summarized in Supplementary Table 1.

### Identification of pyroptosis characterizations in colon cancer

The pyroptosis-related genes were derived from published studies. In order to identify distinct pyroptosis characterization clusters in colon cancer, we used ConsensuClusterPlus R package to execute consensus clustering based on the mRNA expression of these pyroptosis-related genes. Consensus clustering is repeated 1000 times to ensure the stability of molecular classification [39].

### Functional annotation analysis

The Gene Set Variation Analysis (GSVA) and GO enrichment analysis was used to execute functional annotation for uncovering the signaling pathways involved in these pyroptosis characterizations. The “c2.cp.kegg.v6.2.symbols” gene set was downloaded from GSEA database [40–42]. The limma package was utilized to identify differentially expressed genes (DEGs) between three clusters. The DEGs with P value < 0.05 was considered as significant [43]. We then conducted GO enrichment analysis to reveal the biological pathways associated with these DEGs [44].

### Inference of TME cell abundance

Multiple algorithms have now been developed for assessing the abundance of various immune cell infiltrates of the tumor microenvironment based on gene expression. In this study, we used single sample gene set enrichment analysis (ssGSEA) to measure the abundance of microenvironmental immune cell infiltration under three pyroptosis characterizations. We obtained the gene sets with 28 immune cells from a previous study [45, 46].

### Establishment of pyroptosis related signature (PyroSig)

Considering that pyroptosis characterizations played an important role in the prognosis and microenvironment of colon cancer, we constructed pyroptosis related signature. First, a univariate Cox regression model was used to explore the correlation between patient prognosis and the expression of these DEGs. The least absolute shrinkage and selection operator regression method (LASSO) was then performed for the expression of the DEGs with the prognosis P value less than 0.001 [47]. The PyroSig signature was defined as follows:


$$\text{PyroSig}=\sum_{i=1}^{n}\text{Coefi}*Expri$$

where i is the expression of prognosis-related genes.

### Statistical analysis

We used the Kaplan-Meier method to plot survival curves and used the log-rank tests for significance test based on the survminer R package. In addition, a univariate and multivariate Cox regression models were used to reveal the prognostic value of PyroSig signature as a continuous variable. The MaxStat R package was utilized to determine the optimal cut-off point of PyroSig to classify patients into low and high groups. We executed the Wilcoxon test to calculate the difference significance between two groups. The One-way ANOVA test and Kruskal-Wallis tests were utilized to calculate the difference significance groups of three or more [48]. All data analyses were handled based on the software R 4.0.5. All statistical P-values were two-sided, with a statistical significance of p < 0.05.

### Data availability statement

All data related to this work can be acquired from the Gene-Expression Omnibus (GEO, http://www.ncbi.nlm.nih.gov/geo) and the GDC portal (https://portal.gdc.cancer.gov/).

## Supplementary Material

Supplementary Figure 1

Supplementary Tables
