# Supplementary material for: Molecular subtypes identified by pyroptosis-related genes are associated with tumor microenvironment cell infiltration in colon cancer
Source: Aging (Albany NY). 2022 Nov 16;14(22):9020–36. doi: 10.18632/aging.204379 (PMC9740378; doi:10.18632/aging.204379)
Supplement: Supplementary Figure 1 [file aging-14-204379-s001.pdf]

SUPPLEMENTARY FIGURE

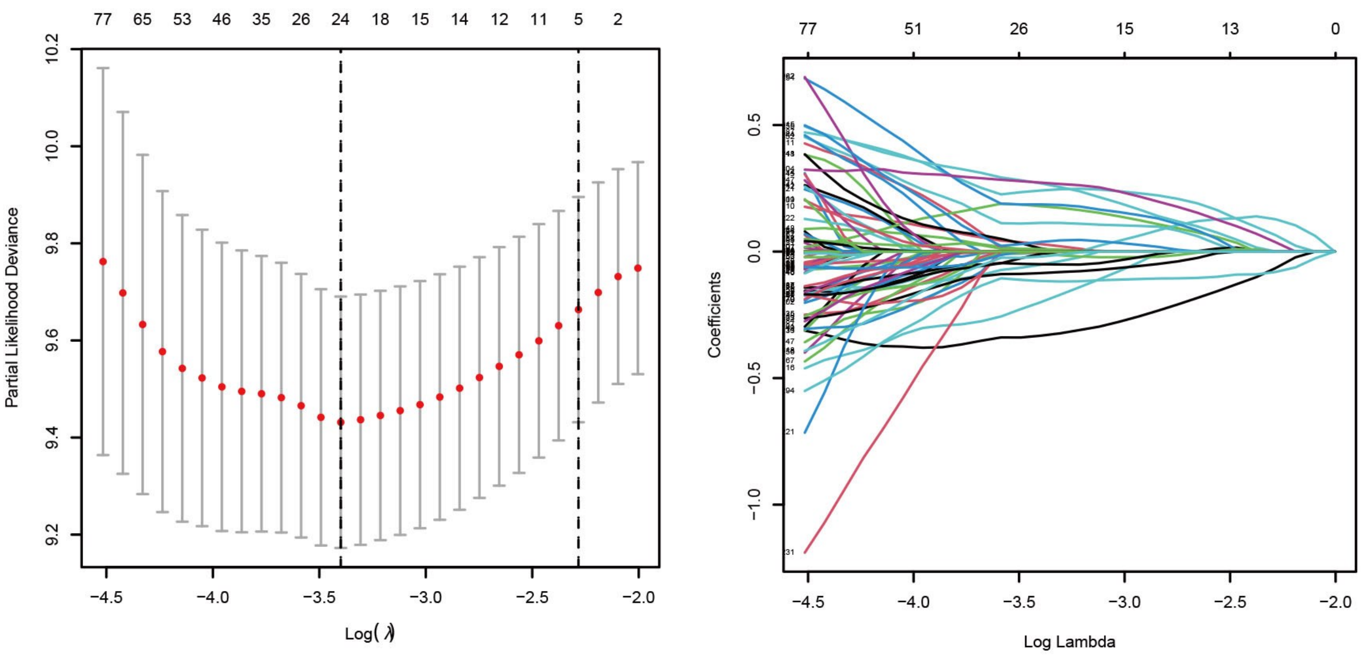

Supplementary Figure 1. Identifying signature genes in colon cancer based on the LASSO model to construct PyroSig signature.
