# Supplementary material for: Molecular subtypes identified by pyroptosis-related genes are associated with tumor microenvironment cell infiltration in colon cancer
Source: Aging (Albany NY). 2022 Nov 16;14(22):9020–36. doi: 10.18632/aging.204379 (PMC9740378; doi:10.18632/aging.204379)
Supplement: Supplementary Tables [file aging-14-204379-s002.pdf]

SUPPLEMENTARY TABLES

Supplementary Table 1. The basic information of datasets included in this study.

Source	Platform	Number of patients	Survival
TCGA-COAD	Illumina RNAseq	514	OS
GSE39582	Affymetrix Human Genome U133 Plus 2.0 Array	585	RFS/OS
GSE17536	Affymetrix Human Genome U133 Plus 2.0 Array	177	RFS
GSE37892	Affymetrix Human Genome U133 Plus 2.0 Array	130	RFS
GSE38832	Affymetrix Human Genome U133 Plus 2.0 Array	122	RFS

Supplementary Table 2. The LASSO coefficient of the signature genes.

Gene	Coefficient
SGK1	0.029851098
POFUT1	-0.200520959
APOL6	-0.332489247
CCL13	0.00429936
PEAR1	0.113373389
TPM4	0.241256572
MCC	0.001070563
CCDC88C	-0.052312181
MYB	-0.006091323
RPP14	-0.004724394
ZNF57	-0.085188108
MLLT3	-0.003590551
HOXA4	0.179238763
MARVELD2	-0.049983419
ZNF564	-0.000432386
GDI1	0.013007788
ETV5	0.186508141
ORAI3	0.269976608
DTX3L	-0.009202089
WWC3	0.010115175
ANKRD32	-0.116197339
RGCC	0.272613636
PLD3	0.03784518
DENND2D	-0.019782871
